# FEELING VALUED IN OLD AGE: A QUALITATIVE EXPLORATION OF THE SOCIAL STATUS IN LATER LIFE

**DOI:** 10.1093/geroni/igad104.2189

**Published:** 2023-12-21

**Authors:** Tim Kuball, Chiara Hohaus, Georg Jahn

**Affiliations:** Chemnitz University of Technology, Chemnitz, Sachsen, Germany; Chemnitz University of Technology, Chemnitz, Sachsen, Germany; Chemnitz University of Technology, Chemnitz, Sachsen, Germany

## Abstract

Subjective social status describes a person’s self-assessed position in a social hierarchy and is an important determinant for mental and physical health. One’s perceived position is dynamically changing across the life-span. Individuals attempt to achieve and preserve status in their social environment. Negative age stereotypes are fueled by and contribute to the attribution of lower social status to older adults. However, little research relates to how status changes and potential status losses are subjectively perceived in older adulthood. In the present study, we used semi-structured interviews to qualitatively identify factors contributing to older adults’ experienced and anticipated status developments. In addition, resources and strategies that are used to maintain relative social status in old age were openly explored. Our sample of 14 East German citizens (mean age = 73.85, range: 67–81 years) reported a highly differentiated understanding of status that included not only socioeconomic factors but also social participation. Experienced changes in status were connected to societal upheavals and retirement. Autonomy and health were reported as important prerequisites for relative status-level preservation. Furthermore, new personal freedoms after retirement entailed empowering opportunities via engagement and other leisure activities for many respondents. Findings implicate that status development in old age may diverge from societal and loss-oriented status expectations if structural support for individual social participation is enabled. In our discussion, we reflect on suitable methodology for studying the construct of social status in old age.

